# Prevalence of depressive symptoms among elderly in the city of Tremembé, Brazil: preliminary findings of an epidemiological study

**DOI:** 10.1590/S1980-57642013DN70300004

**Published:** 2013

**Authors:** Karolina G. César, Leonel T. Takada, Sonia M.D. Brucki, Ricardo Nitrini, Luiz Fernando C. Nascimento, Maira O. Oliveira, Camila M.S. Gomes, Milena C.S. Almeida, Fábio H. Porto, Mirna L.H. Senaha, Valéria S. Bahia, Mônica S. Yassuda, Thaís B.L. Silva, Jéssica N. Ianof, Lívia Spíndola, Magali T. Schmidt, Mário S. Jorge, Patrícia H.F. Vale, Mário A. Cecchini, Luciana Cassimiro, Roger T. Soares, Márcia Rúbia Gonçalves, Ana Caroline S. Martins, Elisângela Rocha, Patrícia Daré

**Affiliations:** 1 MD, PhD Students, Department of Neurology, University of São Paulo. Cognitive and Behavioral Neurology Unit, Department of Neurology, University of São Paulo.; 2 MD, PhD. Cognitive and Behavioral Neurology Unit, Department of Neurology, University of São Paulo.; 3 MD, PhD, Full Professor. Professor of Neurology, University of São Paulo Medical School. Cognitive and Behavioral Neurology Unit, Department of Neurology, University of São Paulo.; 4 MD, PhD. Professor at University of Taubaté.; 5 Tremembé Epidemiologic Study (TES) Group, Department of Neurology, University of São Paulo.

**Keywords:** depression, elderly, prevalence

## Abstract

Depression is a heterogeneous mental disease classified as a set of disorders, which manifest with a certain duration, frequency and intensity. The prevalence of depression in the elderly ranges from 0.5 to 16%.

**OBJECTIVE:**

To establish, in an epidemiological study, the prevalence of significant depressive symptoms in the population aged 60 years or older.

**METHODS:**

Results of a cross-sectional epidemiological study, involving home visits, being carried out in the city of Tremembé, Brazil, were reported. The sample was randomly selected by drawing 20% of the population over 60 years from each of the city's census sectors. In this single-phase study, the assessment included clinical history, physical and neurological examination, cognitive evaluation, the Cornell scale and the Patient Health Questionnaire for psychiatric symptoms. Scores greater than or equal to 8 on the Cornell scale were taken to indicate the presence of depressive symptoms.

**RESULTS:**

A total of 455 elders were assessed, and of these 169 (37.1%) had clinically significant depressive symptoms (CSDS). Depression prevalence was higher among women (p<0.001) and individuals with lower education (p=0.033). The Chi-square test for trends showed a significant relationship where lower socioeconomic status was associated with greater likelihood of depressive symptoms (p=0.005).

**CONCLUSION:**

The prevalence of depressive symptoms was high in this sample of the population-based study and was associated with female gender, low educational level and socioeconomic status. The assessment of the entire population sample must be completed.

## Abstract (pt)

Depressão é uma doença mental heterogênea classificada como um conjunto de transtornos, que se manifestam numa certa duração, frequência e intensidade. A prevalência de depressão em idosos varia de 0,5 a 16%.

**OBJETIVO:**

estabelecer a prevalência de sintomas depressivos significantes em estudo epidemiológico em população acima de 60 anos.

**MÉTODOS:**

Estudo epidemiológico do tipo transversal, no qual estão sendo realizadas visitas domiciliares na cidade de Tremembé, Brasil. A amostra foi aleatória, através do sorteio de 20% da população acima de 60 anos de cada setor censitário do município. Este estudo é de única fase, sendo realizada anamnese, exames físico e neurológico, avaliação cognitiva e aplicação de escalas de Cornell e questionário Patient Health Questionnaire para verificar sintomas psiquiátricos. Foi adotado como critério da presença de sintomas depressivos, pontuação maior ou igual a 8 na escala de Cornell.

**RESULTADOS:**

Foram avaliadas 455 pessoas e destas 169 (37,1%) apresentaram sintomas depressivos significativos clinicamente (SDSC). A maior prevalência foi entre as mulheres (p<0,001) e com escolaridade mais baixa (p=0,033). Quando realizado o teste de qui-quadrado de tendência, houve relação significativa, à medida que diminui o nível socioeconômico, aumenta a chance da presença de sintomas depressivos (p=0,005).

**CONCLUSÃO:**

A prevalência de sintomas depressivos foi elevada nesta amostra do estudo populacional e com associação com gênero feminino, baixo nível educacional e socioeconômico, mas há necessidade de finalizar toda amostragem.

## INTRODUCTION

Depression is a major and growing public health problem and is believed to be a leading cause of mental disability.^1^ People with depression experience symptoms such as feelings of sadness, decreased energy, lack of confidence, negativity, and changes in sleep and appetite; depression is also a predictor of decline in functional abilities.^2,3^

Depression is a heterogeneous mental illness that encompasses a group of disorders, which present with a certain duration, frequency and intensity and are caused by genetic, biochemical, psychological and social/familial factors.^4^

The prevalence of depression in the elderly population worldwide ranges from 0.5% in China to 13.8% in the Dominican Republic.^5^ The combined prevalence of significant depressive symptoms and major depressive disorder in elderly Brazilians was found to be 7% in São Paulo^6^ while depression in the general population was 16.1% in Porto Alegre.^2^

The aim of this study was to establish the prevalence of significant depressive symptoms in an epidemiological study conducted to diagnose cognitive disorders in the population aged 60 years or older in the city of Tremembé, state of São Paulo, Brazil.

## METHODS

This study is a cross-sectional epidemiological study, in which home visits are being carried out in the city of Tremembé. The city is located in the state of São Paulo, about 140 km from the state capital. According to the population census conducted by the Brazilian Institute of Geography and Statistics (IBGE) in 2011, the city had a population of 40,751 inhabitants, of whom 3,690 were aged 60 years or older.^7^

**Sampling.** The initial parameters of the sample were calculated for a study of the prevalence of cognitive impairment with and without dementia, which constituted the primary objective of this study.

We randomly chose 20% of the population over 60 years from each of the city's sectors, as defined by the census, to obtain a homogeneous representation of all regions and districts encompassing all socioeconomic and cultural levels within the city. According to the IBGE there are 89 sectors (73 urban and 16 rural) in the city, and participants were chosen based on a list also provided by the IBGE. Seven hundred and thirty individuals aged 60 years or older were then randomly selected.

Following the selection, letters were sent by mail with an invitation to participate in the study. Subsequently, a community agent visited the households and scheduled a home visit, if the subjects agreed. The subjects or legal guardians were fully informed about the study and signed a consent form.

Only one individual was included from each selected household. Individuals institutionalized in either of the city's nursing homes were also included in the study following randomization, but not all individuals in these institutions were examined, only the individuals randomly selected as part of the 20% of their respective census sector.

**Exclusion criteria.** The study excluded only those who refused to participate, as well as those who did not have informants to answer the questionnaires. When the selected elder refused to participate, we sought to invite the nearest neighbor aged 60 years or older to take part instead, in order to minimize sample loss and try to maintain the percentage of seniors sampled from each sector.

Census sectors that housed any of the city's prisons were excluded from the study (sectors 9, 27 and 54). Five sectors (23, 24, 40, 43 and 46) were excluded because population counts and ages in these regions were presumed (no address and/or ages were confirmed by the census). Another three sectors (53, 68 and 76) were excluded for being expansion areas with no residents.

**Assessment.** This is single-phase study, and therefore history-taking, physical and neurological examination, cognitive assessment, psychiatric evaluation and functional activity questionnaires were done in a single visit.

Cognitive assessment included the following tests: Brief Cognitive Screening Battery (BCSB), Addenbrooke's Cognitive Examination - Revised (ACE-R) and the Montreal Cognitive Assessment (MoCA). The BCSB assesses naming, learning of 10 line figures and delayed recall, and also includes semantic verbal fluency and the clock drawing test.^8^ The ACE-R is an instrument with high sensitivity and specificity for detecting mild dementia, and includes the Mini Mental State Examination (MMSE).^9,10^ The MoCA can be applied relatively quickly and has been used to detect mild cognitive impairment, even with normal performance on MMSE.^11^ For functional evaluation, the IQCODE (Informant Questionnaire on Cognitive Decline in the Elderly)^12^ with 16 items and the Functional Activities Questionnaire were used.^13^

For the evaluation of psychiatric symptoms two scales were employed: the Cornell depression scale^14,15^ and Patient Health Questionnaire (PHQ) from the Primary Care Evaluation of Mental Disorders (PRIMEMD).^16,17^ The Cornell Scale, although initially developed for the diagnosis and monitoring of depression in patients with dementia, is a validated instrument for both demented and non-demented geriatric subjects.^18^ It is also slightly more complete than the geriatric depression scale,^19^ also covering issues related to anxiety, behavioral and sleep changes. The PHQ is useful for the diagnosis of mood disorders, anxiety disorders, somatoform disorders, disorders related to alcohol, and eating disorders; and besides presenting validity similar to the original PRIME-MD, it is considered more efficient.^16^

A score greater than or equal to 8 points on the Cornell scale was adopted as a diagnostic criterion for clinically significant depressive symptoms (CSDS). As there is no consensus in the literature on the PHQ cutoff score for diagnosis of depression or other psychiatric disorders, scores greater than or equal to 5 "yes" were taken to indicate somatic complaints and mood disorders; however, at this preliminary stage of the study it was decided not to use this to diagnose CSDS.

Socioeconomic classification was performed based on a recent classification by the Brazilian Association of Market Research (ABIPEME).^20^

**Statistical analysis.** Statistical analysis was performed using SPSS (Statistical Package for the Social Sciences) version 17.0. The degree of association between depressive symptoms and age, level of education, and socioeconomic status was determined by Pearson's Chi-square test for the crossed variables. Epi-Info was used to evaluate the association between depressive symptoms and increasing age, education, and socioeconomic level whereas the Chi-square test was employed for linear trends. Significance was set at p<0.05.

The project and the free and informed consent form were approved by the University of São Paulo Research Ethics Committee (protocol 0378/09).

## RESULTS

This study is still ongoing and, out of the initial planned sample of 730 subjects, 455 participants have been evaluated to date. Of this total, only five selected participants were residents of nursing homes. Table 1 shows the sample's demographic data.

**Table 1:** Sociodemographic characteristics of the study sample.

Variables		N	Percentage %
Age group	60-64 years	107	23.5
	65-69 years	113	24.8
	70-74 years	91	20.0
	75-79 years	68	14.9
	80-84 years	43	9.5
	≥85 years	33	7.3
Sex	Male	168	36.9
	Female	287	63.1
Years of education	Zero/illiterate	42	9.2
	1-4 years	258	56.7
	5-8 years	70	15.4
	9-11 years	44	9.7
	≥12 years	41	9.0
Socioeconomic level*	A1	1	0.2
	A2	16	3.5
	B1	43	9.4
	B2	102	22.3
	C1	133	29.0
	C2	90	19.7
	D	69	15.1
	E	1	0.2

* ABIPEME: Brazilian Association of Market Research.

Mean age was 71.19 (±7.91) years with a slight predominance of women (62.7%). Mean years of education was 5.08 (±4.29) years. White individuals were the majority, representing 75.8% of the total; whereas 23.1% were of African descent (18.9% brown and 4.2% black) and only 1.1% were of Asian descent.

Of the total number of subjects evaluated, 207 (45.5%) had subjective memory complaints. Of these, 101 (59.7%) also had depressive symptoms.

A score greater than 5 points on the PHQ was found for 265 (58.4%) of the subjects examined, showing a significant prevalence of somatic complaints such as headaches, spine and joint-related pains, changes in bowel and digestive habits, sleep problems, among others. The mean PHQ score was 5.74 (±3.78), the minimum score was zero and the maximum 18, with a variance of 14.32.

Participants who had a score on the Cornell scale greater than or equal to eight were considered to have CSDS and represented 37.1% of the population studied(169 participants).

Depressive symptoms were more prevalent among individuals aged 85 years or over (48.5% of this age group); however, there was also a high prevalence among younger elderly aged 60-64 years (41.1% of individuals in this age group). There was no tendency for increase in the presence of depressive symptoms with advancing age (p=0.92). There was no significant association between CSDS and age (p=0.528).

We found significant associations between gender and CSDS (p<0.001), or with education (p=0.033). The prevalence of CSDS was higher among women and individuals with lower educational levels. The higher the educational level, the lower the tendency of presenting with depressive symptoms (p=0.013).

No significant association was found between CSDS and socioeconomic status (p=0.112) on the Chi-square test, but when the Chi-square test for linear trend was used an association between lower socioeconomic status and greater chance of depressive symptoms (p=0.005) was found. The highest prevalence was in class D (46.4%) and class E, in which only one individual evaluated had depressive symptoms. These results are given in Table 2.

**Table 2:** Analysis of the association between depressive symptoms and sociodemographic characteristics.

Variables		N	Depressive symptoms	OR (95%CI)	P value
Age group					0.528
	60-64 years	107	44	1.00	0.92
	65-69 years	113	41	0.82	
	70-74 years	91	31	0.74	
	75-79 years	68	22	0.68	
	80-84 years	43	15	0.77	
	≥85 years	33	16	1.35	
Sex					<0.001
	Male	168	42	2.4 (1.54-3.73)	
	Female	287	127		0.033
Years of education					
	Zero/illiterate	42	19	1.00	0.013
	1-4 years	258	98	0.74	
	5-8 years	70	32	1.02	
	9-11 years	44	12	0.45	
	≥12 years	41	8	0.29	
Socioeconomic level*					0.112
	A1+A2	17	3	1.00	0.005
	B1+B2	145	43	1.97	
	C1+C2	223	90	3.16	
	D	69	32	4.04	

* ABIPEME: Brazilian Association of Market Research. Level E was not included because it contained only one participant.

## DISCUSSION

In this study, we found a prevalence of 37.1% of depressive symptoms among elderly in the city of Tremembé, Brazil. The prevalence was higher than that reported by other studies conducted in Brazil (whose rates ranged from 6.4 to 26.9% in the SABE study,^21^ 16.1% in the city of Porto Alegre^2^ and 13% in the city of Sao Paulo,^6,22^ but the caveat is that we assessed depressive symptoms rather than diagnosed the participants with depression.

The sample size was calculated to establish the prevalence of cognitive impairment with and without dementia which ranges from 7% to 20%, and therefore includes the prevalence of depression in the elderly which is between 7% and 16%. Hence, we do not believe the sample size was a factor impacting the high prevalence of depressive symptoms found in this population. This high prevalence may be explained by missing yet-to-beevaluated individuals of higher socioeconomic status, given the sample is not yet complete.

The higher prevalence of depressive symptoms in this study can also be explained by evidence from previous studies showing that elderly individuals tend to have a lower prevalence of major depressive disorders but a higher prevalence of CSDS, compared to young adults. This can be explained by the fact that symptoms in this population may or may not be related to cognitive deficits, sensorineural hearing loss, and/or medical co-morbidities with polypharmacy.^23-25^

Recent publications have shown significant variability in the presence of depressive symptoms among individuals with mild cognitive impairment, ranging from 3% to 63%, and some research has suggested depression can mark a continuum for cognitive impairment initially without, and later with, dementia.^26,27^

In the present study, the Cornell Scale was used to detect the prevalence of depressive symptoms. Even though some studies have shown the scale can be used in patients with and without dementia, and that scores above 8 define a diagnosis of depression,^14,15,18^ there is controversy regarding this score and we therefore chose not to diagnose patients with depression, but rather report the presence of depressive symptoms.

The higher prevalence of CSDS in women is in accordance with previous studies.^6,22,24,28^ This can be explained by the fact that women more readily take on their psychological problems and talk more openly about them,^29^ and even accept, more so than men, to participate in population-based studies.

No significant association was found between age and depressive symptoms, a result differing from data reported by previous studies showing that the greater the age, the greater the occurrence of depression.^2,23-25,27^ There was a higher depression prevalence in this study among participants aged 85 years or older, but there was also a high prevalence among younger participants. It is possible the age-depression association was not found because the full study sample has yet to be assessed.

The association between depressive symptoms and low educational levels is in agreement with data from the literature.^2,30,31^ Subjects with higher levels of education are better informed, and have better access to health care; and this would also explain the literature data showing higher prevalence of depressive symptoms among those with lower socioeconomic levels – a finding also observed in this sample when evaluated for trend. However, Barcelos-Ferreira et al.^22^ failed to find this association in the city of São Paulo, perhaps explained by the fact that even individuals with lower socioeconomic and educational levels can nowadays gain access to information.

In conclusion, these preliminary results for the study population showed a high prevalence (37.1%) of depressive symptoms, and an association with female gender, low educational level and socioeconomic status. As this is a single-phase study, patients with depressive symptoms were not reevaluated to confirm a diagnosis of depression or otherwise. All patients with memory complaints shall be followed, and participants with depressive symptoms could also be followed to allow diagnostic clarification, evaluation of the accuracy of the Cornell scale and PHD, as well as determine the correlation between depression and cognitive decline.

**Study support.** Supported by FAPESP grant number 2012/04815-6. Study conducted in the city of Tremembé, state of São Paulo, Brazil by the Department of Neurology, University of São Paulo Medical School.
